# Identification of Cytochrome b‐245, beta‐chain gene mutations, and clinical presentations in Iranian patients with X‐linked chronic granulomatous disease

**DOI:** 10.1002/jcla.23637

**Published:** 2020-10-23

**Authors:** Atefeh Heydari, Farhad Abolnezhadian, Mahnaz Sadeghi‐Shabestari, Alihossein Saberi, Ahmad Shamsizadeh, Ata A. Ghadiri, Pegah Ghandil

**Affiliations:** ^1^ Cellular and Molecular Research Center Ahvaz Jundishapur University of Medical Sciences Ahvaz Iran; ^2^ Department of Medical Genetics School of Medicine Ahvaz Jundishapur University of Medical Sciences Ahvaz Iran; ^3^ Department of Pediatrics Abuzar Children's Hospital Ahvaz Jundishapur University of Medical Sciences Ahvaz Iran; ^4^ Immunology research center of Tabriz‐TB and lung research center of Tabriz‐children hospital Tabriz University of Medical Sciences Tabriz Iran; ^5^ Infectious and Tropical Diseases Research Center Health Research Institute Ahvaz Jundishapur University of Medical Sciences Ahvaz Iran; ^6^ Department of Immunology Cellular and Molecular Research Center School of Medicine Ahvaz Jundishapur University of Medical Sciences Ahvaz Iran; ^7^ Diabetes Research Center Health Research Institute Ahvaz Jundishapur University of Medical Sciences Ahvaz Iran

**Keywords:** *CYBB* gene, immunodeficiency, mutation, patients, X‐linked chronic granulomatous disease (X‐CGD)

## Abstract

**Background:**

X‐linked chronic granulomatous disease (X‐CGD) is an immunodeficiency disorder caused by defects in the gp91^phox^ subunit that leads to life‐threatening infections. We aimed to identify *CYBB* gene mutations and study clinical phenotypes in Iranian patients with probable X‐CGD.

**Methods:**

We studied four unrelated Iranian patients with probable X‐CGD and their families recruited in several years. We isolated genomic DNA from whole blood and performed Sanger sequencing in the *CYBB* gene's coding and flanking regions. We also performed pathogenicity predictions using in silico tools.

**Results:**

We detected four different mutations, including a novel insertion mutation in exon 5 (p.Ile117AsnfsX6), in the patient. Bioinformatics analysis confirmed the pathogenic effect of this mutation. We predicted protein modeling and demonstrated lost functional domains. The patient with the insertion mutation presented pneumonia and acute sinusitis during his life. We also detected three other known nonsense mutations (p.Arg157Ter, p.Arg226Ter, and p.Trp424Ter) in the *CYBB* gene. The patient with p.Arg157Ter developed lymphadenitis and pneumonia. Moreover, the patient with inflammatory bowel disease showed p.Arg226Ter and the patient with tuberculosis presented p.Trp424Ter. We detected different clinical features in the patients compared to other Iranian patients with the same mutations.

**Conclusion:**

Our results expand the genetic database of patients with X‐CGD from Iran and make it much easier and faster to identify patients with X‐CGD. Our results also help to detect carriers and enable prenatal diagnosis in high‐risk families as a cost‐effective strategy.

## Graphical abstract

We studied four Iranian patients suspected to X‐linked chronic granulomatous disease and their families. One novel and three known mutations were detected. The clinical features of them have been presented. The pathogenic effect of the novel mutations was confirmed by bioinformatics analysis.

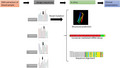

## 1 INTRODUCTION

Chronic granulomatous disease (CGD) (OMIM #306400) is a primary immunodeficiency described by the total absence or low degrees of reactive oxygen species (ROS) generation. ROS is a result of a defect in the nicotinamide adenine dinucleotide phosphate oxidase (NADPH oxidase) complex in phagocytes. Chronic granulomatous disease is a rare disorder with repeated and life‐threatening bacterial and fungal infections that involves different organs such as lymph nodes, gastrointestinal tract, and lungs.^1^ It is accompanied with pneumonia, abscess, and granuloma formation in response to chronic inflammation.^2^, ^3^

Mutations in five different genes can cause CGD with autosomal recessive or X‐linked recessive inheritance patterns. *CYBA, NCF1, NCF2*, and *NCF4* genes situated on an autosomal chromosome encode structure segments of NADPH oxidase protein (p22^phox^, p47^phox^, p67^phox^, and p40^phox^, respectively). Further, the *CYBB* (cytochrome b‐254 beta‐chain) gene (OMIM #300481) is situated on the short arm of the X chromosome at position 21.1, containing 13 exons and spanning 30 kb. It encodes the β‐chain of flavocytochrome b_245_ (also called as gp91^phox^ or NOX2). It is a necessary component of the NADPH oxidase complex in phagocytes, such as granulocytes, monocytes, and macrophages.

The gp91^phox^ protein encoded by the *CYBB* gene comprises an N‐terminal domain and a C‐terminal domain. N‐terminal is anticipated to form six transmembrane α‐helices, and C‐terminal is a cytoplasmic domain containing binding sites for flavin adenine dinucleotide (FAD) and NADPH. These domains are necessary for transferring electrons to phagosomes.^4^ The *CYBB* gene is created by a wide variety of mutations, including deletions, splice site mutations, and missense or nonsense mutations.^5^, ^6^, ^7^, ^8^, ^9^

According to the published data from the United States, European countries, and Japan, the most well‐known inherited pattern was X‐linked chronic granulomatous disease (X‐CGD) (≥65%). Nevertheless, among CGD patients in North Africa, the Arab world, Turkey, and Iran, the autosomal recessive inheritance pattern is more frequent. These results highlight the diverse inherited pattern and a high rate of consanguinity marriages.^10^, ^11^, ^12^, ^13^, ^14^

Several studies reported mutations of the *CYBB* gene in CGD patients with clinical symptoms. Jose Antonio Tavares de Albuquerque et al. (2018) found a novel mutation in the *CYBB* gene in a male Brazilian patient with pneumonia.^15^ Teimourian et al. (2018) characterized four new mutations in the *CYBB* gene in 10 Iranian families.^16^ Harvindar Kaur Gill et al. (2013) examined a Chinese X‐CGD patient with prolonged fever and recurrence of lymphadenitis in the axillary lymph node. They found a nonsense mutation in the *CYBB* gene.^17^ Sun Hi Ko et al. (2014) screened 26 Korean families with recurrent infections and found eight novel mutations in the *CYBB* gene.^4^ Marie Jose´ Stasia et al. (2005) detected six novel mutations and five reported mutations in the *CYBB* gene of 11 French patients with sepsis, pneumonia, infectious dermatitis, and recurrent or severe abscess.^9^ This study aimed to identify *CYBB* gene mutations in Iranian patients suspected to have X‐CGD and examine the relationship between these mutations and the patients' phenotype.

## 2 MATERIALS AND METHODS

### 2.1 Ethics statement

The ethics committee of Ahvaz Jundishapur University of Medical Sciences approved this study (IR.AJUMS.REC.1397.853). Informed consent was received from all patients and their families enrolled in the study.

### 2.2 Patients

In this study, we enrolled four unrelated kindreds with patients suspected to have X‐CGD for several years. The patients were identified through a clinical interview in the pediatric immunodeficiency clinic at the Abuzar Children's Hospital, at a primary referral pediatric hospital in the southwest of Iran, and at a children's hospital in Tabriz in the north of Iran.

We selected young male patients based on clinical symptoms (frequent infections) confirmed using clinical tests. The patients were positive for the nitroblue tetrazolium (NBT) test and the dihydrorhodamine 123 (DHR 123) assay.

### 2.3 Description of patients and kindreds

#### 2.3.1 Family 1

**FIGURE 1:**
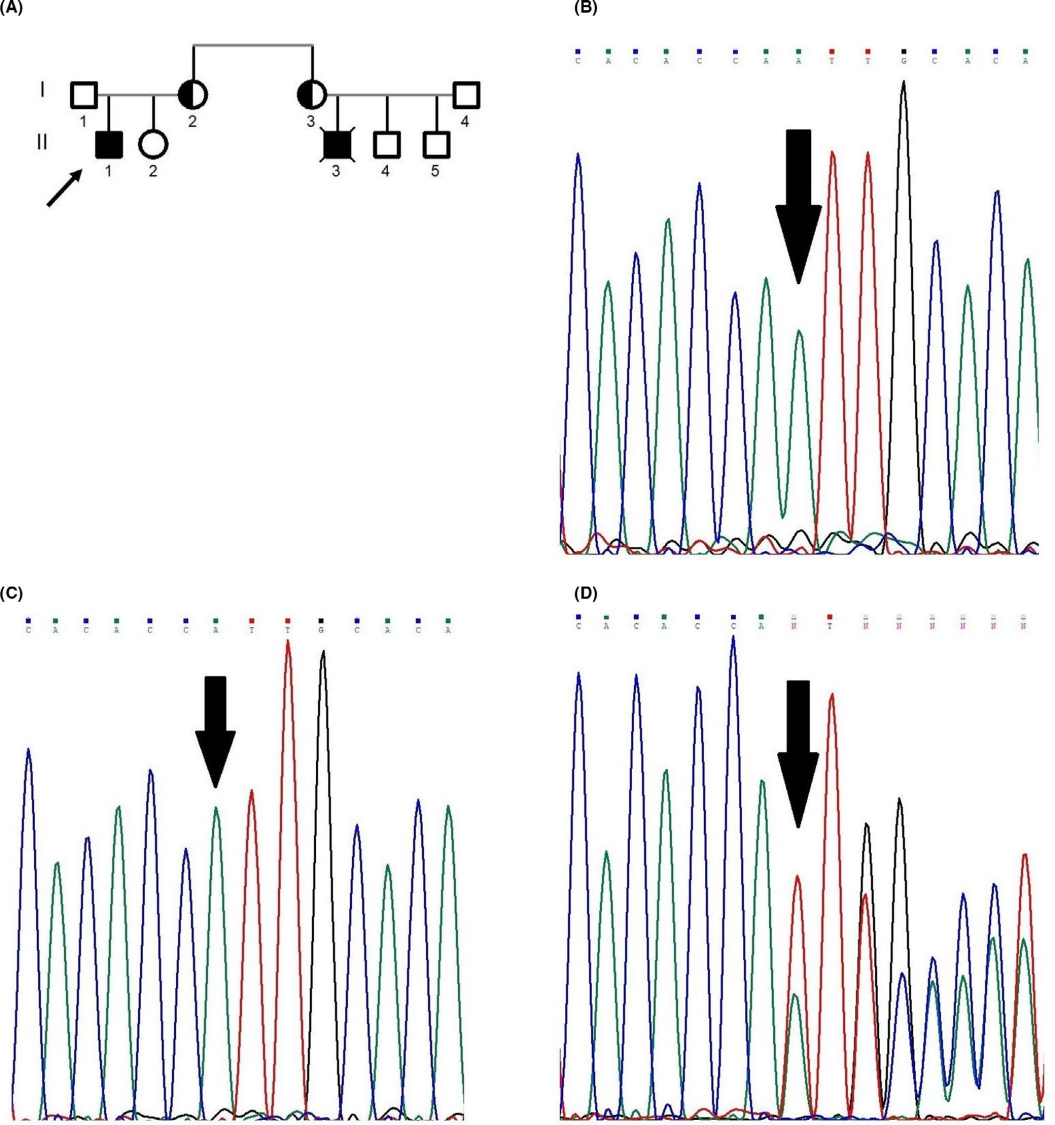
A, The pedigree of the first studied family; B and C, the sequence chromatograph of the mutation c.348‐349insA in the patient 1 with hemizygous state and the wild‐type sequence in the *CYBB* gene; D, the sequence analyses of the patient's mother with heterozygous state for the mutation c.348‐349insA

In the first family with Bakhtiari ethnicity, the proband's cousin (Figure 1,II3) was suspected to have X‐CGD based on the NBT and DHR tests and his clinical phenotype. He died when he was 8 years old because of pneumonia. No precise information was available on him. Following this history, the proband's mother (Figure 1,I2) was suspected to be an X‐CGD carrier based on the NBT test. The proband (Figure 1,II1), born in 2008, was diagnosed to have X‐CGD at birth based on the NBT (2 %) and DHR (5 %) tests. He was admitted to a hospital for pneumonia when he was 8 years old and responded to the treatment. After that, acute sinusitis was diagnosed in him several times and then was treated.

#### 2.3.2 Family 2

In the second family with Fars ethnicity, the proband, born in 2009, was diagnosed with severe lymphadenitis and pneumonia when he was 3 months old. He was suspected to have X‐CGD based on the NBT (no positive cell) and DHR (2.16 %) tests. Then, he was hospitalized several times due to pneumonia. Finally, he died at the age of eight despite taking preventative medicine. Furthermore, he had an older brother who died of severe fever when he was 40 days before the proband was born.

#### 2.3.3 Family 3

In the third family with Turkish ethnicity, the proband, born in 2017, was diagnosed with chronic gastritis and colitis when he was 10 months old. He was suspected to have X‐CGD based on the NBT (no positive cell) and DHR (1.3 %) tests. He was often diagnosed with inflammatory bowel disease (IBD) during his life, although he responded to the treatment and currently has a healthy life. He also had an older brother who died during the childhood because of IBD.

#### 2.3.4 Family 4

In the fourth family with Kurdish ethnicity, the proband, born in 2001, was diagnosed with the frequent abscess when he was 1 year old. He was suspected to have X‐CGD based on the NBT (no positive cell) and DHR (2.6 %) tests. A few years later, he underwent surgery due to spinal tuberculosis (TB) when he was nine. He responded well to the operation and medication.

### 2.4 Sequencing and mutational analysis

Genomic DNA was isolated from the EDTA whole blood of the patients and their families using the YEKTA TAJHIZ kit (cat. No: FABGK001).

All 13 exons and exon‐intron boundaries of the *CYBB* gene were amplified by polymerase chain reaction (PCR) with conditions and primers described previously.^18^ Sanger sequencing and data analysis were performed for all PCR products in the patients using an ABI Prism 3700 apparatus (Big Dye Terminator sequencing kit, Applied Biosystems). Meanwhile, all available mothers and siblings of the patients with the detected mutation were analyzed. We used several databases such as ENSEMBL (https://www.ensembl.org/index.html), HGMD (http://www.hgmd.cf.ac.uk/), and dbSNP (https://www.ncbi.nlm.nih.gov/snp/) to investigate whether the detected variants were novel or were previously reported as a pathogenic mutation or polymorphism. Furthermore, we used Mutation Taster (http://www.mutationtaster.org/) to anticipate the pathogenic role of the identified mutation.

Moreover, sequence alignment was carried out using the ENSEMBLE database and Clustal‐w for a new mutation to examine the area in distinct species.

We named new variants based on guidelines of the human genome variation society using human CYBB protein sequences with the reference ENSG00000165168.

Moreover, we used the SWISS‐MODEL Web site (https://swissmodel.expasy.org/) to predict the structure of wild type and mutant protein and recognize the position of newly identified mutations in mutant protein. The I‐Mutant 3.0 Web site (http://gpcr2.biocomp.unibo.it/cgi/predictors/I‐Mutant3.0/I‐Mutant3.0.cgi) was used to calculate the stability of mutant protein compared with normal protein by measuring ΔΔG through the structure‐based mode. Moreover, a crystal structure of the wild‐type gp91^phox^ NADPH binding domain was obtained from the protein data bank (PDB 3A1F).

The nonsense‐mediated mRNA decay (NMD) ESC predictor Web site (https://nmdprediction.shinyapps.io/nmdescpredictor/) was used to predict whether a frameshift variant would escape NMD.

## 3 RESULTS

We identified a novel insertion mutation (c.348‐349insA) located on exon 5 of the *CYBB* gene with the hemizygous state for the proband of the first family (Figure 1B). This mutation was a frameshift. The amino acid at position 122 was converted to stop codon and create premature stop codon (p.Ile117AsnfsX6). The pathological effect of the mutation was examined

by in silico analysis (Mutation Taster) (Table 1). The mutation is predicted as a disease‐causing agent and is not reported in 1000 genome databases. The patient's mother and sister (Figure 1A,I2,II2) were analyzed. The patient's healthy sister was the wild type for this region (Figure 1C), while his mother was heterozygote for this mutation as a carrier (Figure 1D).

**Table 1:** The data of Mutation Taster Tool about the effect of the mutation c.348‐349insA on the patient 1

Summary	NMD
	Amino acid sequence changed
	Frameshift
	Protein features (might be) affected
	Splice site changes
**Analyzed issue**	**Analysis result**
Name of alteration	No title
Alteration (Phys, location)	chr23:37652928_37652929insA
Alteration type/region	Insertion/CDS
AA changes	I117Nfs*6
Position(s) of altered AA	117 (frameshift or PTC—further changes downstream)
Frameshift	yes
Length of protein	NMD
Position (AA) of stop codon in wt/mu AA sequence	571/122
Wild‐type AA sequence	MGNWAVNEGLSIFVILVWLGLNVFLFVWYY RVYDIPPKFFYTRKLLGSALALARAPAACL NFNCMLILLPVCRNLLSFLRGSSACCSTRV RRQLDRNLTFHKMVAWMIALHSAIHTIAHL FNVEWCVNARVNNSDPYSVALSELGDRQNE SYLNFARKRIKNPEGGLYLAVTLLAGITGV VITLCLILIITSSTKTIRRSYFEVFWYTHH LFVIFFIGLAIHGAERIVRGQTAESLAVHN ITVCEQKISEWGKIKECPIPQFAGNPPMTW KWIVGPMFLYLCERLVRFWRSQQKVVITKV VTHPFKTIELQMKKKGFKMEVGQYIFVKCP KVSKLEWHPFTLTSAPEEDFFSIHIRIVGD WTEGLFNACGCDKQEFQDAWKLPKIAVDGP FGTASEDVFSYEVVMLVGAGIGVTPFASIL KSVWYKYCNNATNLKLKKIY FYWLCRDTHAFEWFADLLQLLE SQMQERNNAGFLSYNIYL TGWDESQANHFAVHHDEEKDVITGLKQKTL YGRPNWDNEFKTIASQHPNTRIGVFLCGPE ALAETLSKQSISNSESGPRGVHFIFNKENF*
mutated AA sequence	MGNWAVNEGLSIFVILVWLGLNVFLFVWYY RVYDIPPKFFYTRKLLGSALALARAPAACL NFNCMLILLPVCRNLLSFLRGSSACCSTRV RRQLDRNLTFHKMVAWMIALHSAIHTNCTSI*

Note

All positions are in base pairs (bp) if not explicitly stated differently.

AA, amino acid; CDS, coding sequence; mu, mutated; NMD, nonsense‐mediated mRNA decay; nt, nucleotide; wt, wild‐type.

According to the American College of Medical Genetics guideline,^19^ the sequence variant (c.348‐349insA) in the *CYBB* gene is classified as a pathogenic variant due to having the pathogenic very strong criterion (PVS1, a null variant in a gene where LOF is a known mechanism of disease) and the pathogenic moderate criterion (PM2, absent from controls in Exome Sequencing Project, 1000 Genomes Project, or Exome Aggregation Consortium).

**FIGURE 2:**
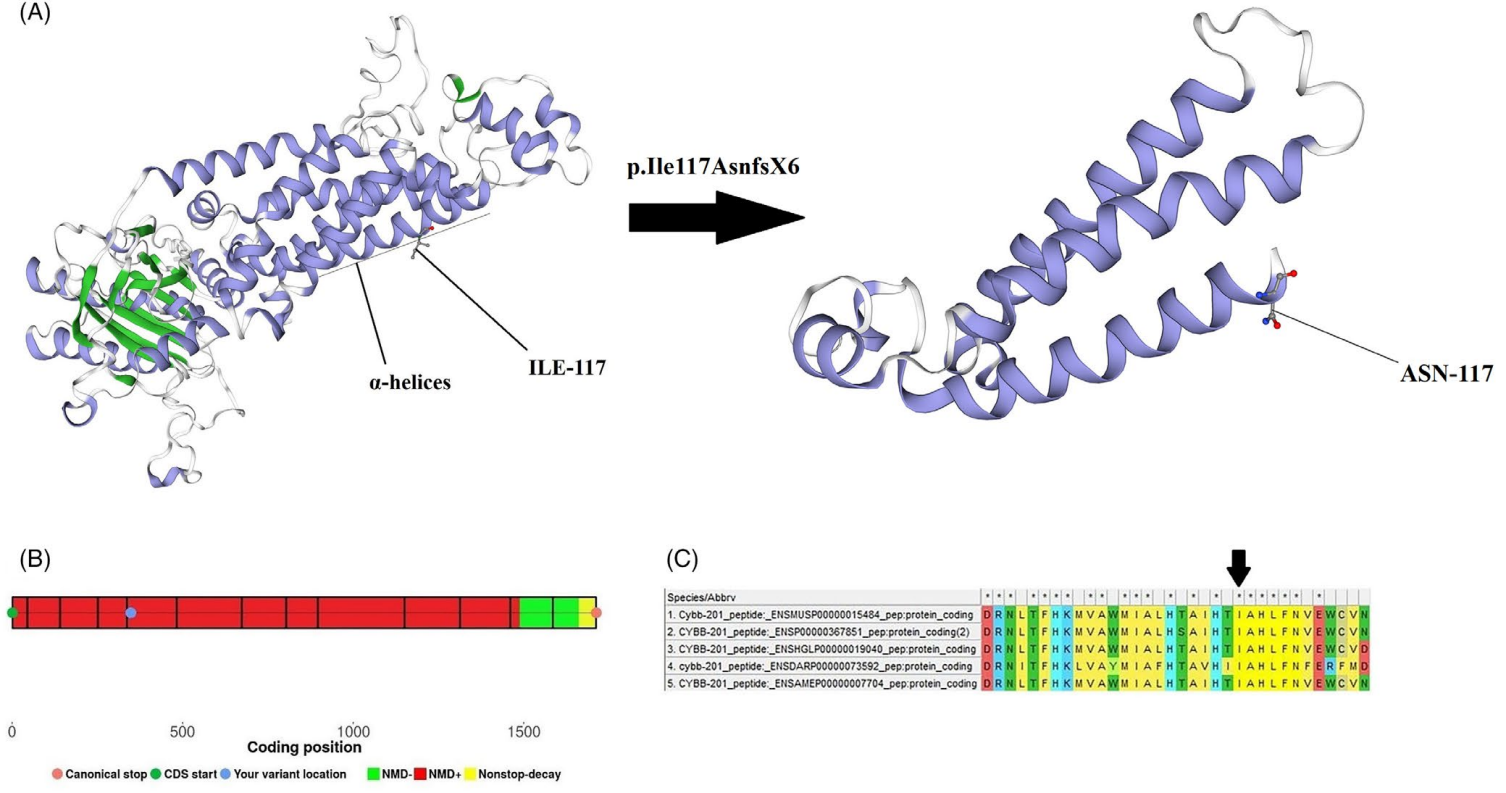
A, The structure of normal and mutant gp91^phox^ designed by the SWISS‐MODEL website in the patient 1; B, the result of nonsense‐mediated mRNA decay prediction in the patient 1 by the NMD Esc Predictor Web site; C, the alignment of the *CYBB* gene sequence in five different species by Clustal‐w, showing that this mutation is in a conserved sequence

Based on Mutation Taster Server, this insertion mutation is located between amino acids 103‐123, which represents the transmembrane α‐helices of gp91^phox^. The SWISS‐MODEL server was used for structural and functional analysis and revealed that this mutation caused loss of some parts of the subunit in the structure of gp91^phox^ (Figure 2A). Moreover, the NMD prediction Web site predicted that this premature stop codon caused NMD (Figure 2B). This region has been conserved among species (Figure 2C).

The I‐Mutant server calculated the free energy changed (ΔΔG) value equal to −1.56 kcal/mol with a reliability index of 6 for this mutation. Therefore, the structural stability of this mutant protein largely decreased.

**FIGURE 3:**
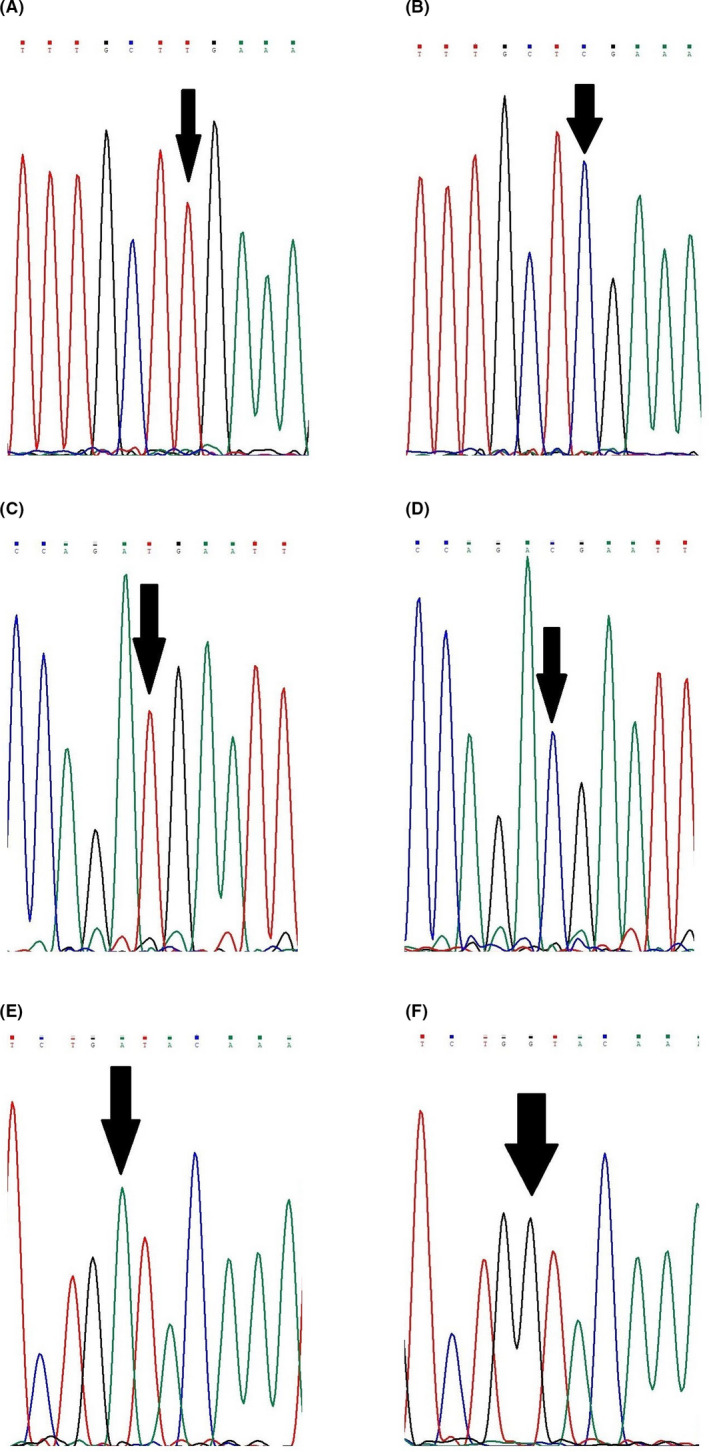
The sequence chromatographs of mutations detected in the patients 2, 3, and 4 with hemizygous state, as well as the wild‐type sequence in the *CYBB* gene; A and B, the mutation c.469C>T in the patient 2 and the healthy control; C and D, the mutation c.676C>T in the patient 3 and the healthy control; E and F, the mutation c.1272G>A in the patient 4 and the healthy control

In the proband of the second family, a known pathogenic nonsense mutation was detected in exon 5 of the *CYBB* gene (c.469C>T) with hemizygous state (Figure 3A,B). The mutation caused the change in arginine to a stop codon (p.Arg157Ter).

In the third family, a known nonsense mutation was found in exon 7 of the *CYBB* gene (c.676C>T) with hemizygous state (Figure 3C,D). The mutation led to the change in arginine to a stop codon (p.Arg226Ter), and it was located at the N‐terminal of gp91^phox^. The patient's mother and sister were heterozygous for this mutation as a carrier.

A known pathogenic nonsense mutation (c.1272G>A) with hemizygous state was found in exon 10 of the *CYBB* gene in the patient 4 (Figure 3E,F). The mutation caused the change in tryptophan to a stop codon (p.Trp424Ter), and it was located at the cytoplasmic domain of gp91^phox^. Furthermore, the patient's mother and sister were heterozygous for the mutation.

## 4 DISCUSSION

In this study, we identified four mutations in four Iranian kindreds with X‐CGD patients and realized that one of the mutations was novel.

We detected a novel frameshift mutation (c.348‐349insA) in exon 5 of the *CYBB* gene in the patient 1. It was located at the transmembrane domain of gp91^phox^, which is a vital site for transferring electrons. Analyses of in silico tools predicted loss of some essential parts of gp91^phox^ and changed protein modeling. These essential parts have a α‐helical structure and are vital for transferring electrons. NMD probably occurred in the patient 1. Even if this transcript escaped NMD in this patient, the resulting protein appears to be truncated to 122 residues instead of the wild‐type protein with 571 residues and will probably be pathogenic and disease‐causing. Base on the I‐Mutant server, this insertion mutation may alter the free energy and subsequently the protein stability. Folding free energy is an essential biophysical feature in proteins, showing the overall stability of the three‐dimensional structure of macromolecules.^20^ We also detected a nonsense mutation (c.469C>T) in exon 5 of the *CYBB* gene that led to Arg157Ter. The pathological effect of the mutation was first reported in 1994 by Ariga in Japan. He examined two different Japanese CGD patients based on clinical symptoms and the NBT test and detected a mutation (c.469C>T) in one of them.^21^ In Iran, Teimourian et al. (2018) reported this mutation in an Iranian boy with abscess and fistula.^16^ However, the patient 2 with (c.469C>T) in our study developed lymphadenitis and pneumonia.

We detected another nonsense mutation (c.676C>T) in exon 7 of the *CYBB* gene. The mutation was reported for the first time by Curnutte in 1992^22^ and by Teimourian in 2008 in Iran.^23^ The reported Iranian patient had skin and liver abscesses, while our patient had IBD.

We identified one other nonsense mutation (c.1272G>A) in exon 10 of the *CYBB* gene in the patient 4. The mutation was described for the first time by Newburger in 1998.^24^ It was also reported in an Iranian patient in 2008^23^ and a Korean patient in 2014.^4^ Iranian patients with (c.1272G>A) presented disease with bloody diarrhea, cervical lymphadenitis, and pneumonia. Our patient with the same mutation developed an abscess and spinal TB.

Furthermore, Jacinta Bustamante et al. indicated that respiratory burst in human macrophages is a crucial mechanism for protective immunity to tuberculous mycobacteria.^25^ Several studies have been published on X‐CGD patients with TB. For example, Cecilia Barese et al. (2004) screened a group of patients with pulmonary tuberculosis and unusual complications in Argentina. They detected seven novel mutations in the *CYBB* gene in these patients.^26^ Bustamante et al. (2007) revealed a French 12‐year‐old boy with dispersed tuberculosis. They detected a mutation in the *CYBB* gene of the patient, leading to diagnosis of X‐CGD.^27^ Taj Ali Khan et al. (2016) detected a novel mutation in exon 10 of the *CYBB* gene in a Pakistani patient with Mycobacterium tuberculosis and subcutaneous abscesses in the neck.^28^

All of the mutations found in these articles, and other X‐CGD patients with TB are different from the mutation detected in the patient 4 (c.1272G>A) in the current study. Movahedi et al. (2004) examined the clinical features of Iranian CGD patients. It appears that patients with CGD might be susceptible to tuberculosis infections, and thus, tuberculosis should be properly prevented in all of them.^3^ Generally, as it has been mentioned, pneumonia is the most well‐known infection experienced in CGD patients in all age groups. The patients 1 and 2 in our study also had pneumonia during their life.

Analysis of the nonsense mutations in our patients showed that two out of the three mutations were C>T ([c.469C>T], [c.676C>T]) and the remaining one was G>A (c.1272G>A) (C>T on the antisense). Moreover, Teimourian et al. analyzed nonsense mutations obtained in their study and suggested that the methylation‐induced deamination of cytosine may be a significant mechanism for nonsense X‐CGD mutations in Iran.^23^

Roos et al. listed 681 distinct mutations in the *CYBB* gene and examined their frequency. They reported that deletion (35.6%) and missense (21.3%) mutations were the most common mutations found in the *CYBB* gene, with 7.9% of these mutations being insertion and 14.1% of them being nonsense.^29^ We reported an insertion mutation and three nonsense mutations in this article, with approximately low frequency in the *CYBB* gene.

In Iran and countries with consanguineous marriages, X‐CGD is rare to compare to the autosomal recessive form. For this reason, few studies have been performed on the Iranian population.

## 5 CONCLUSION

Our data could be helpful to early diagnose patients suspected to have X‐CGD, and also, to provide correct clinical diagnosis and optimize therapeutics in these children such as medication, bone marrow transplantation, and gene therapy. Furthermore, carrier screening in X‐CGD patients' families could be beneficial for early diagnosis and prevention of X‐CGD in children born with the disease. Meanwhile, these findings can expand the genetic database of *CYBB* gene mutations; however, conducting further research on X‐CGD patients will be necessary.

## AUTHOR'S CONTRIBUTIONS

Farhad Abolnezhadian performed and supervised the clinical studies in the patients. He wrote and supervised clinical parts of the manuscript. Mahnaz Sadeghi‐Shabestari and Ahmad Shamsizadeh performed and supervised the clinical studies in the patients. Alihossein Saberi and Ata A. Ghadiri planned the experiments. Pegah Ghandil and Atefeh Heydari planned and carried out the experiments. Atefeh Heydari wrote the first draft of the manuscript with support from Pegah Ghandil. Pegah Ghandil conceived and developed the presented idea, supervised the experiments, and wrote and supervised the manuscript.

## Data Availability

The data supporting the study findings are available from the corresponding author upon reasonable request.
